# Supplementary material for: Temporal transcriptomic profiling of human three-dimensional neuromuscular co-cultures
Source: Biol Open. 2025 Sep 16;14(9):bio062196. doi: 10.1242/bio.062196 (PMC12486213; doi:10.1242/bio.062196)
Supplement: Supplementary information [file biolopen-14-062196-s1.pdf]

Table S1. 3 vs 1

Available for download at
https://journals.biologists.com/bio/article-lookup/doi/10.1242/bio.062196#supplementary-data

Table S2. 6 vs 3

Available for download at
https://journals.biologists.com/bio/article-lookup/doi/10.1242/bio.062196#supplementary-data

Table S3. 3vs1 6vs3 padj005

Available for download at
https://journals.biologists.com/bio/article-lookup/doi/10.1242/bio.062196#supplementary-data

Table S4. GO Terms - Group 1

Available for download at
https://journals.biologists.com/bio/article-lookup/doi/10.1242/bio.062196#supplementary-data

Table S5. GO Terms - Group 2

Available for download at
https://journals.biologists.com/bio/article-lookup/doi/10.1242/bio.062196#supplementary-data

Table S6. GO Terms - Group 3
Available for download at
https://journals.biologists.com/bio/article-lookup/doi/10.1242/bio.062196#supplementary-data

Biology Open • Supplementary information

Table S7. GO Terms - Group 4

Available for download at
https://journals.biologists.com/bio/article-lookup/doi/10.1242/bio.062196#supplementary-data

Table S8. GO Terms - Group 5

Available for download at
https://journals.biologists.com/bio/article-lookup/doi/10.1242/bio.062196#supplementary-data

Table S9. GO Terms - Group 6

Available for download at
https://journals.biologists.com/bio/article-lookup/doi/10.1242/bio.062196#supplementary-data

Table S10. GO Terms - Group 7

Available for download at
https://journals.biologists.com/bio/article-lookup/doi/10.1242/bio.062196#supplementary-data

Table S11. GO Terms - Group 8

Available for download at
https://journals.biologists.com/bio/article-lookup/doi/10.1242/bio.062196#supplementary-data

Biology Open • Supplementary information
